# Advances in the Total Synthesis of Angular Triquinane-Type Natural Products

**DOI:** 10.3390/molecules30193956

**Published:** 2025-10-02

**Authors:** Xin Wang, Dunfeng Liu, Yunfei Cheng

**Affiliations:** School of Chemistry and Chemical Engineering, Huangshan University, Huangshan 245061, China

**Keywords:** angular triquinane, Pauson–Khand reaction, tandem cyclization, skeletal reorganization

## Abstract

This review provides a comprehensive summary of advances in synthetic strategies for structurally complex angular triquinane natural products, with a particular emphasis on terpenoids and alkaloids over the past two decades. The formidable challenge inherent in these frameworks lies in the stereoselective construction of the congested, angularly fused triquinane core bearing one or more quaternary stereocenters. We systematically categorize and critically evaluate the key methodologies employed to construct this central motif, highlighting four major strategic approaches: (i) The Pauson–Khand reaction; (ii) tandem or cascade cyclization processes; (iii) skeletal reorganization strategies; and (iv) other innovative methods, including ring-expansion, radical cyclizations, and photochemical pathways.

## 1. Introduction

**Figure 1:**
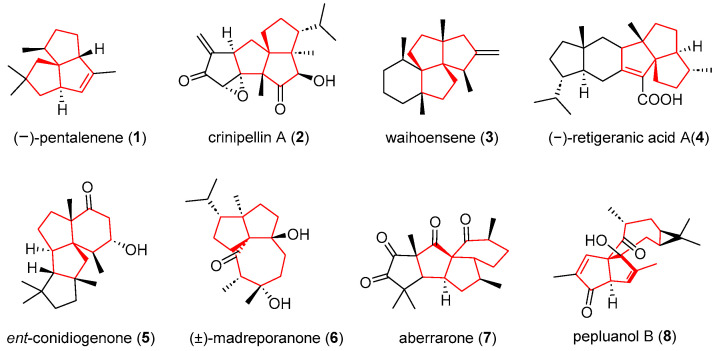
Representatives of angular triquinane-type natural products.

Angular triquinanes refer to tricyclic frameworks sharing a quaternary carbon vertex, typically with 5-5-5, 5-6-5, or 5-7-5 ring fusion topologies. These structurally intricate systems are prevalent in a wide array of bioactive natural products, including terpenes, alkaloids, and lactones, and exhibit diverse biological activities such as antibacterial, anticancer, anti-inflammatory, and antioxidant properties [1,2,3,4,5,6,7,8,9,10,11,12,13,14,15]. As shown in Figure 1, crinipellin A (**2**), isolated from the basidiomycete *Crinipellis stipitaria*, features an angular 5-5-5 tricyclic carbocycle and demonstrates significant antibacterial and anticancer activities [9,10]. Aberrarone (**7**) was tested against the most common etiologic agent of malaria, Plasmodium falciparum (W-2 chloroquine-resistant strain) [12]. Pepluanol B (**8**) has good inhibitory activity on the KV1.3 potassium ion channel, with an IC_50_ value of 9.50 μM [14,15]. Additional angular triquinane derivatives have also been reported to exhibit anti-inflammatory, antimicrobial, and antioxidant activities, further underscoring the therapeutic potential of this compound class [16,17,18,19,20].

A major hurdle in synthesizing angular triquinanes stems from the presence of one or more contiguous quaternary carbon centers, which significantly increases structural complexity and poses substantial challenges for synthetic chemists. Conventional stepwise transformations are often insufficient for efficiently constructing these congested polycyclic scaffolds, necessitating the development of sophisticated synthetic strategies. To address this gap, this review focuses on four major synthetic approaches for accessing angular triquinanes: the Pauson–Khand reaction, skeletal reorganization strategies, radical tandem cyclization reactions, and diverse cycloaddition reactions and ring-rearrangement metathesis. For each strategy, we critically discuss its unique advantages, limitations, and representative applications in natural product synthesis.

## 2. Pauson–Khand Annulation: Forging the Core via Metal-Catalyzed Cycloaddition

**Figure 2:**
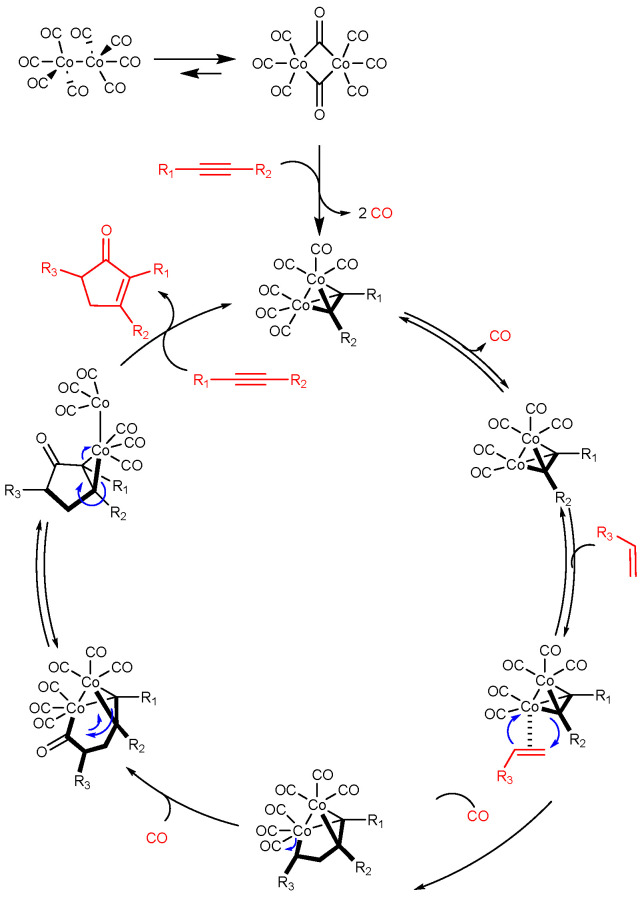
General mechanism of the intramolecular Pauson–Khand reaction.

The Pauson–Khand reaction is a [2 + 2 + 1] cycloaddition of an alkyne, an alkene and carbon monoxide (Figure 2). This reaction is highly valuable for constructing complex structures, particularly cyclopentenones, and allows for better control of stereochemistry owing to its intramolecular nature.

**Scheme 1:**
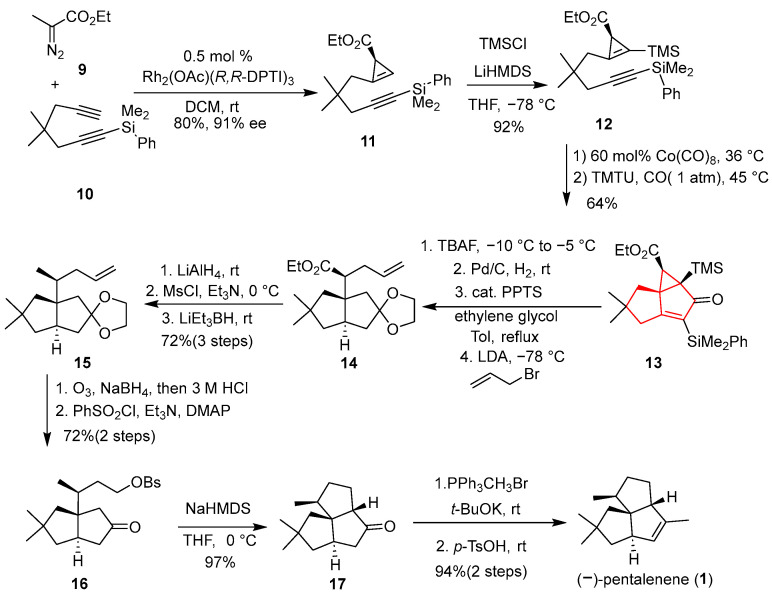
Synthesis of (−)-pentalenene by Fox’s group [21].

In 2007, Fox’s team reported the first enantioselective synthesis of (−)-pentalenene, employing a cascade intramolecular Pauson–Khand reaction catalyzed by noble metal rhodium as the key step to construct a [5,5,3] tricyclic framework [21]. As shown in Scheme 1, ethyl diazoacetate (**9**) underwent stereoselective cyclopropenylation with diyne compound **10** under the catalysis of divalent rhodium to deliver compound **11,** with a yield of up to 80% and an enantioselectivity of 91% ee. Subsequently, the introduction of the TMS group facilitated the Pauson–Khand reaction, resulting in the formation of tricyclic compound **13**. Following seven steps, including deprotection, cyclopropane opening, and introduction of side chains, provided compound **15**, which was converted to ketone **16** via two-step conversion. Under the action of strong base NaHMDS, an intramolecular S_N_2 reaction occurred with high yield, and the tricyclic compound **17** was constructed. Then asymmetric total synthesis of sesquiterpenes (−)-pentalenene (**1**) was accomplished through a Wittig reaction and an isomerization reaction.

**Scheme 2:**
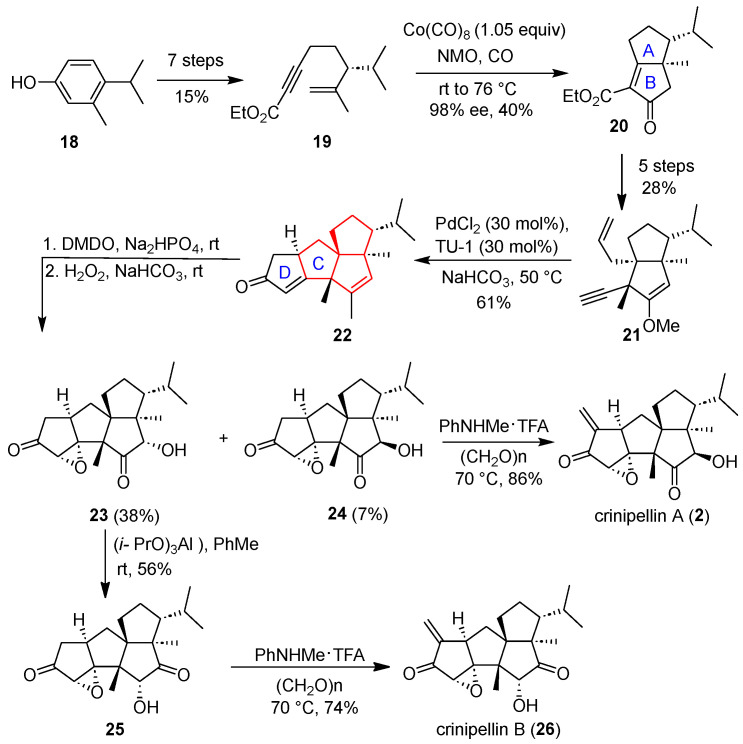
Synthesis of crinipellin A and crinipellin B by Yang’s group [22].

In 2018, Yang’s group reported a strategy for diastereoselective construction of the tetraquinane core of diterpene compounds crinipellin A and crinipellin B via two Pauson–Khand reactions promoted by octacarbonyl cobalt and thiourea/palladium chloride, respectively [22]. As shown in Scheme 2, converting commercially available phenol **18** into alkynyl ester **19** was successful through seven steps, with a 15% yield. Compound **19** underwent the first Pauson–Khand reaction under the action of Cobalt carbonyl, constructing the A and B rings of the diterpene and producing cyclopentenone **20**. A series of functional group conversions of **12** were carried out to obtain alkynyl compound **21**. Under the catalysis of thiourea/palladium chloride [23], the second Pauson–Khand reaction occurred to provide tetracyclic compound **22**. In this case, the C and D rings of the target natural product were constructed in one step. After subsequent adjustments of oxidation state and functional groups of **23** and **24**, asymmetric total synthesis of crinipellin B (**26**) and crinipellin A (**2**), respectively, was achieved. Notably, this strategy will be helpful in the collective synthesis of crinipellin analogs, since it allows the protocol developed to be used to synthesize tetraquinane cores with different substituents through tactical changes in substituents and functionalities.

**Scheme 3:**
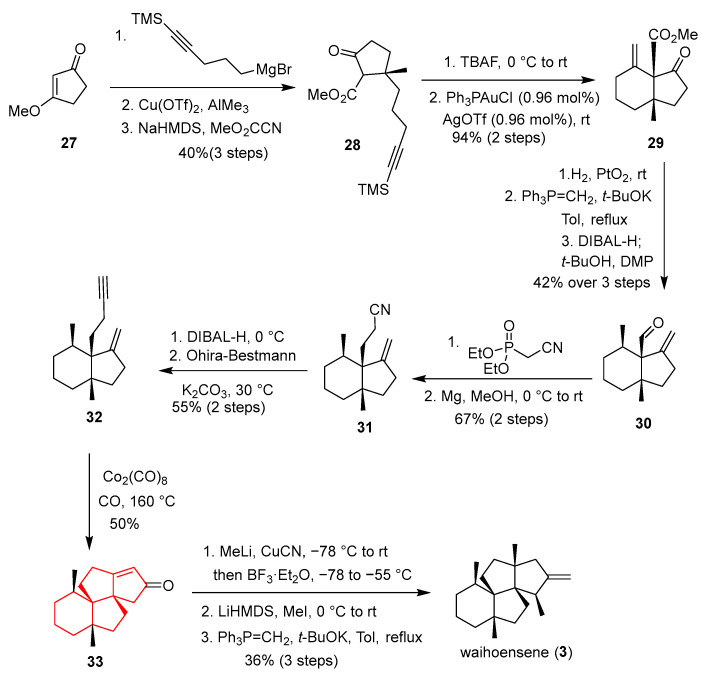
Synthesis of waihoensene by Snyder’s group [24].

In 2020, Snyder’s group reported a highly concise, enantioselective total synthesis of the angular triquinane natural product (+)-waihoensene, a molecule featuring four contiguous all-carbon quaternary centers [24] (Scheme 3). Compound **27** was elaborated to ester **28**, which served as the direct precursor for a pivotal gold-catalyzed Conia-ene reaction (Ph_3_PAuCl, AgOTf), forging a six-membered ring and the second vicinal quaternary center to provide the bicyclic framework **29** in quantitative yield. The key transformation was a challenging intramolecular Pauson–Khand reaction of enyne **32** (Co_2_(CO)_8_, CO, mesitylene, 160 °C), which successfully constructed the third ring and the final quaternary carbon center of the angular triquinane core, delivering intermediate **33**. The synthesis was concluded by installing the fourth quaternary center via a cuprate-mediated methylation and a final Wittig olefination to yield (+)-waihoensene (**3**). This route is remarkable for its strategic use of minimal functional groups to orchestrate multiple critical C–C bond formations, its high convergency, and its innovative application of a Conia-ene reaction to assemble a vicinal quaternary center pair.

**Scheme 4:**
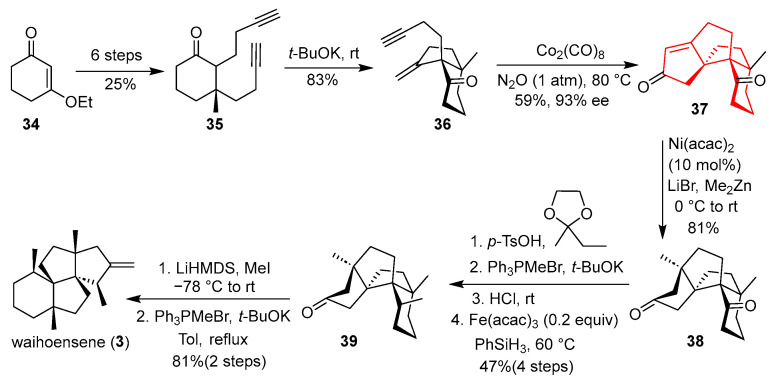
Synthesis of waihoensene by Yang’s group [25].

In 2020, Yang’s group accomplished an asymmetric total synthesis of the highly congested angular triquinane natural product (+)-waihoensene in 15 steps [25] (Scheme 4). The synthesis commenced with enone **34**, which underwent a copper-catalyzed asymmetric conjugate addition and then a Sakurai allylation and ozonolysis to provide the key diyne precursor **35**. The core transformation involved a diastereoselective Conia-ene type cyclization of **35** (*t*BuOK, DMSO, rt) to construct the bicyclic framework **36,** with a new quaternary center. A critical intramolecular Pauson–Khand reaction of enyne **36** (Co_2_(CO)_8_, N_2_O, DCE, 80 °C) then forged the angular triquinane core, delivering tricycle **37** bearing three contiguous all-carbon quaternary centers. A nickel-catalyzed conjugate methylation installed the fourth quaternary center, producing diketone **38**. After ketone protection, Wittig olefination occurred, followed by a highly diastereoselective Fe(acac)_3_-catalyzed hydrogen atom transfer (HAT) reduction (PhSiH_3_, EtOH, 60 °C) to saturate the double bond and set the desired stereochemistry, yielding **39**. Final methylation (LiHMDS, MeI) and Wittig methylenation completed the synthesis of waihoensene (**3**). This route is notable for its high step economy, exceptional diastereoselectivity in critical cyclizations, and the innovative use of a radical HAT process to solve a challenging stereochemical problem.

**Scheme 5:**
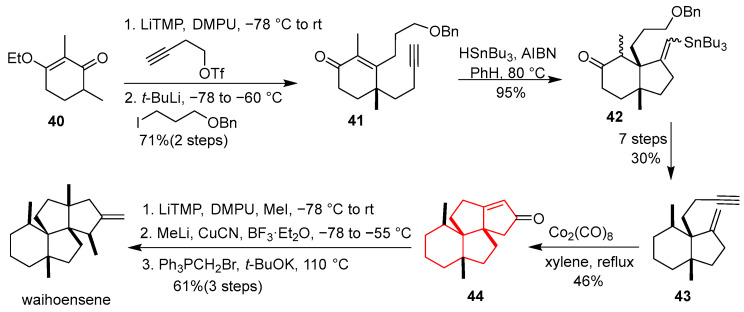
Synthesis of waihoensene by Gaich’s group [26].

In 2021, the Gaich’s group reported the total synthesis of the same molecule waihoensene [26] (Scheme 5). They employed commercially available cyclohexenone derivative **40** as the starting material and utilized aldol addition and an elimination reaction to generate **41**. Under the action of tributyltin hydride and AIBN, a radical cyclization reaction occurred to generate cyclohexanone **42**. Then, through oxidation state adjustment and alkylation reaction, enyne compound **43** was obtained. Under the condition of octacarbonyl cobalt, enyne underwent the key Pauson–Khand reaction to generate **44**. In this way, the four-ring skeleton was constructed, and the total synthesis of waihoensene was completed by following the reference process in the literature within three steps.

**Scheme 6:**
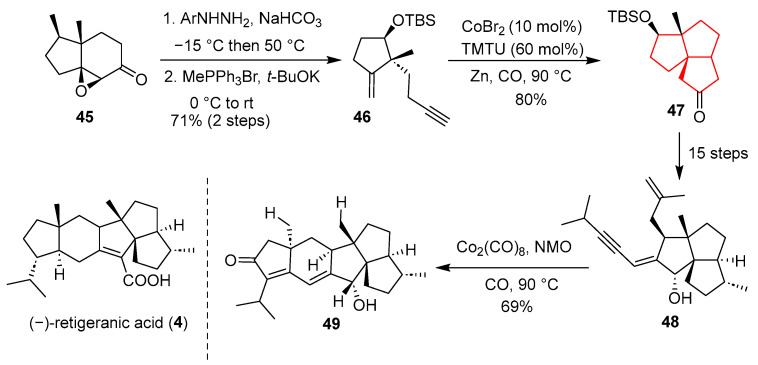
Synthesis of (−)-retigeranic acid by Yang’s group [27].

In 2021, Yang’s group utilized a strategy of two intramolecular Pauson–Khand reactions to synthesize the five-ring core skeleton of sesquiterpene (−)-retigeranic acid [27] (Scheme 6). Starting from the known compound **45**, they synthesized the alkyne compound **46** through Eschenmoser Tanabe fragmentation and a Wittig reaction. The compound underwent the first intramolecular Pauson–Khand reaction under the conditions of cobalt bromide, TMTU, and zinc powder [28] to construct the tricyclic structure **47**. Then, the alkenone **47** underwent a long-step transformation to generate the cyclopentanone **48** with two side chains. Subsequently, the second Pauson–Khand reaction occurred between octacarbonyl cobalt and NMO, completing the construction of the core pentacyclic skeleton of (−)-retigeranic acid (**4**).

The intramolecular Pauson–Khand reaction offers exceptional atom economy and convergence in constructing a cyclopentenone core ubiquitous in angular triquinanes. Its inherent cis-fused stereoselectivity reliably installs vicinal quaternary centers with predictable relative configuration. Recent advances in asymmetric catalysis further enhance enantiocontrol (>90% ee), positioning PKR as a stereodivergent platform for complex terpenoid synthesis. Substrate scope remains constrained to rigid alkyne–alkene tethers, often necessitating protective group manipulations. Toxic Co_2_(CO)_8_ reagents limit scalability, while competing side reactions cap yields at 40–60% for congested systems. Future catalysis designs must address chemo- and regioselectivity in polyunsaturated precursors.

## 3. Skeletal Reorganization Strategies: Structural Editing of Precursor Scaffolds

**Scheme 7:**
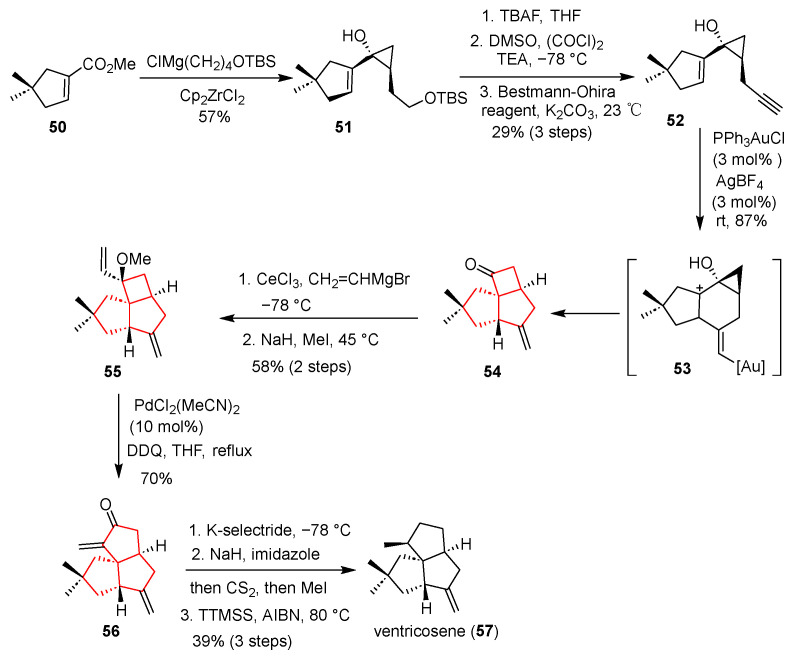
Synthesis of ventricosene by Toste’s group [29].

In 2008, Toste’s team presented an approach for constructing a tricyclic framework by intramolecular cycloisomerization of enynes containing an embedded cyclopropane unit catalyzed by Au (I) [29]. As shown in Scheme 7, Kulinkovich cyclopropanation of vinyl ester **50** was the first step in the process. Three-step functional group conversion of the resulting **51** produced vinyl compound **52**. Gold(I) catalysis facilitated the cycloisomerization of enynes bearing a cyclopropane moiety, selectively generating ring systems with a cyclopropylmethyl cation. Intermediate **53** underwent a Wagner–Meerwein rearrangement, which triggered the diastereoselective formation of fused cyclobutanes to provide compound **54**. Ring-expansion of the corresponding methyl ether **55** in the presence of catalytic PdCl_2_(MeCN)_2_ and DDQ delivered exo-methylene cyclopentanone **56**. Finally, the total synthesis of ventricosene (**57**) was achieved through functional group conversion. It is worth mentioning that the gold-catalyzed ring-expanding enyne cycloisomerizations enabled the efficient construction of architecturally complex polycyclic systems.

**Scheme 8:**
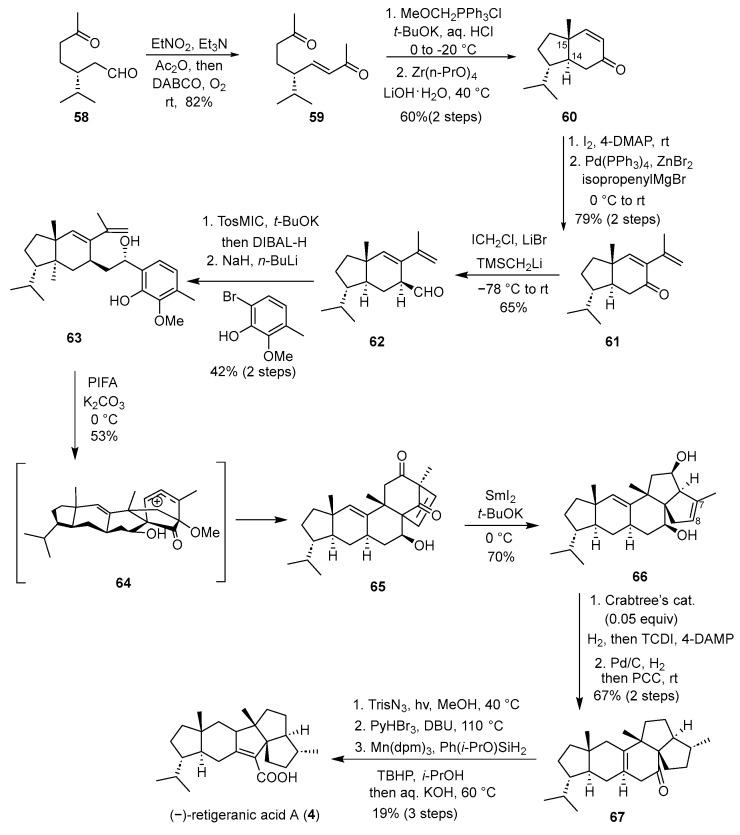
Synthesis of (−)-retigeranic acid A by Ding’s group [30].

In 2023, Ding and co-workers achieved the first asymmetric total synthesis of (−)-retigeranic acid A. A pivotal strategy in their approach featured a reductive skeletal rearrangement cascade, enabling the precise construction of diverse angular triquinane frameworks [30]. As shown in Scheme 8, their approach commenced with a commercially available chiral aldehyde **58**, which underwent a Henry condensation with ethyl nitroethane followed by a one-pot Nef reaction under DABCO/O_2_ to produce enone **59** in 82% yield. Chemoselective Wittig olefination at the C15 ketone, followed by acid hydrolysis, provided a homologated keto-aldehyde. An intramolecular Michael/aldol cyclization mediated by Zr(n-PrO)_4_ then established the trans-hydrindane framework, delivering **60** as the major diastereomer. Subsequent conversion to an α-iodoenone and Negishi coupling with isopropenylmagnesium bromide yielded keto-diene **61**. A cascade epoxidation/Meinwald rearrangement initiated by ICH_2_Cl and TMSCH_2_Li·LiBr, followed by spontaneous epimerization, furnished aldehyde **62**. Van Leusen homologation with TosMIC and *t*-BuOK, DIBAL-H reduction, followed by 1,2-addition of an organolithium reagent, provided vinylphenol **63**, which underwent an oxidative dearomatization-induced (ODI) [5 + 2] cycloaddition/pinacol rearrangement cascade to form pentacycle **65** as a single isomer. The core angular triquinane system was constructed via a reductive skeletal rearrangement cascade: treatment of **65** with SmI_2_ in *t*-BuOH promoted a Dowd–Beckwith rearrangement followed by an acyloin rearrangement, directly producing alcohol **66,** with a 70% yield. Further elaboration included Crabtree’s catalyst-mediated stereoselective hydrogenation and Chugaev elimination, followed by hydrogenation and isomerization to install the double bond, and PCC oxidation to yield **67**. A Wolff ring contraction via UV irradiation of the diazo precursor and a metal-hydride hydrogen atom transfer (MHAT) reduction using Ph(i-PrO)SiH_2_, followed by saponification, delivered (–)-retigeranic acid A (**4**). This synthesis features a concise and innovative reductive rearrangement to access the angular triquinane core, demonstrating broad applicability for constructing congested polyquinane systems.

**Scheme 9:**
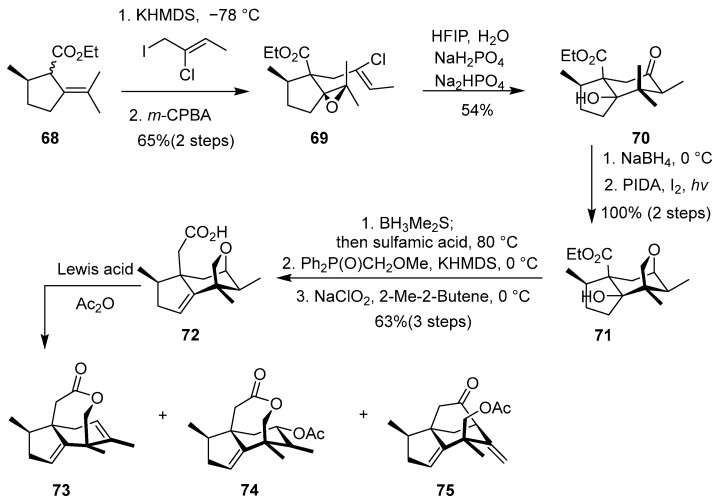
Synthesis of illicium sesquiterpenes by Zhang’s group [31].

In 2023, Zhang and co-workers reported protecting-group-divergent syntheses of illicium sesquiterpenes [31]. As shown in Scheme 9, starting from chiral ester **68**, alkylation with (*Z*)-2-chloro-1-iodobut-2-ene and epoxidation formed epoxide **69**. HFIP-promoted cationic epoxide-ene cyclization afforded bicyclic ketone **70**. NaBH_4_ reduction, Suárez reaction (HAT oxygenation), and homologation gave rapid access to the 15-carbon tricyclic carboxylic acid **72**. Lewis acid (BF_3_·OEt_2_, Bi(OTf)_3_, or TMSOTf)-mediated skeletal reorganization of **72** divergently generated three distinct tricyclic precursors (**73**, **74**, **75**). Subsequent oxidations (e.g., Baeyer–Villiger, dihydroxylation, desaturation) and biomimetic rearrangements yielded eight allo-cedrane/seco-prezizaane sesquiterpenes (e.g., pseudoanisatin in 11 steps, 11-O-debenzoylashironin) and enabled the formal syntheses of five anislactones (e.g., merrilactone A).

**Scheme 10:**
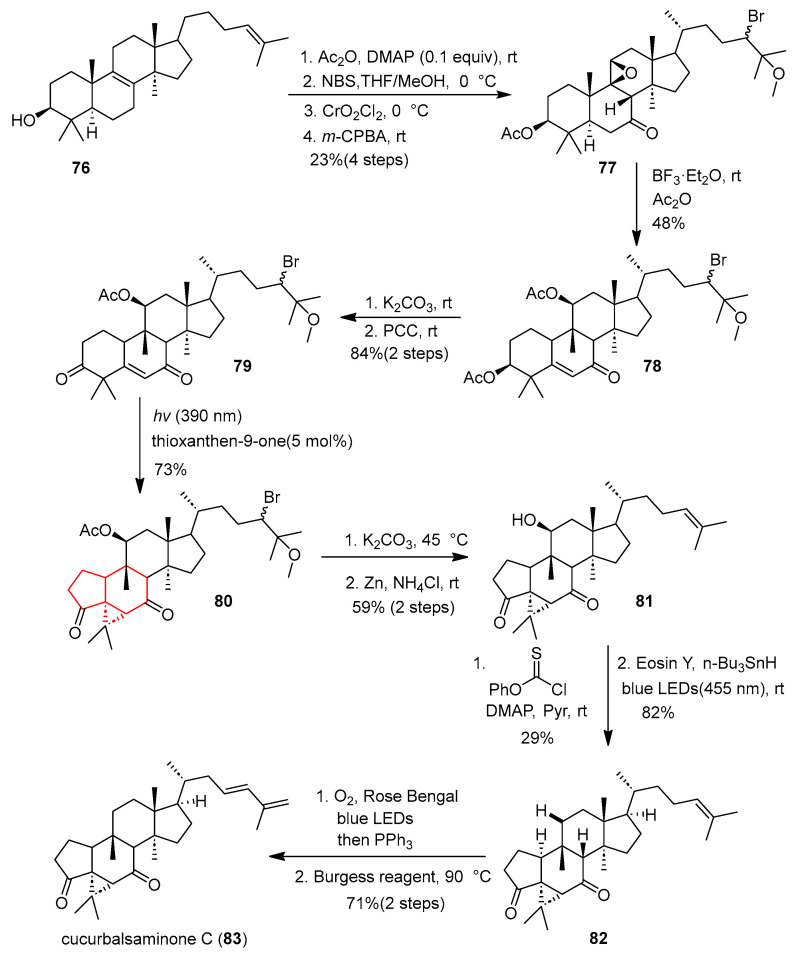
Synthesis of cucurbalsaminone C by Wu’s group [32].

In 2025, Wu’s group accomplished the first total synthesis of *abeo*-cucurbitane triterpenoids cucurbalsaminone C via a bioinspired route featuring two key rearrangements [32]. As shown in Scheme 10, starting from lanosterol **76**, the synthesis firstly installed the cucurbitane skeleton through a biomimetic tandem Wagner–Meerwein rearrangement of epoxide **77** to afford **78**. After oxidation to enone **79**, the pivotal photochemical oxa-di-π-methane (ODPM) rearrangement forged the 5/6/3-fused core **80** with complete diastereoselectivity, overcoming limitations observed in steroid substrates. Subsequent Eosin Y-photocatalyzed Barton–McCombie deoxygenation [(TMS)_3_SiH, blue light] efficiently removed the sterically hindered C11-OH of **81** to give **82**, suppressing competitive Chugaev elimination. Late-stage Burgess dehydration of allylic alcohols **82** furnished cucurbalsaminone C (**83**) (71% over two steps). This synthesis demonstrated the power of photochemical strategies for constructing congested polycyclic scaffolds, though late-stage oxidations remain non-ideal for scalability.

Skeletal editing transcends conventional bond formation logic, enabling the topological metamorphosis of accessible precursors into strained frameworks. Hudlicky’s ring-expansion and Toste’s gold-catalyzed cycloisomerization exemplify how linear or monocyclic substrates undergo concerted reorganization to forge angular triquinanes with >50% yield. Such strategies often mimic biosynthetic pathways, offering step-count advantages. Predictive control over regioselectivity remains elusive due to competing rearrangement pathways. These strategies often require substrates with precise stereoelectronic alignment, and efficient transfer of chiral information remains a challenge. Scalability suffers from stoichiometric reagents (PbCO_3_, SmI_2_) and harsh conditions. Computational modeling of transition states could mitigate these issues.

## 4. Tandem and Cascade Cyclization: Convergent Assembly of the Tricyclic Framework

**Scheme 11:**
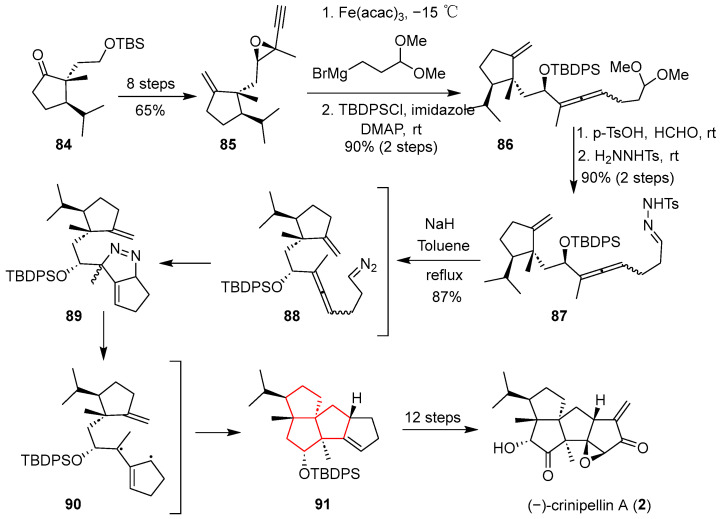
Synthesis of (−)-crinipellin A by Lee’s group [33].

In 2014, the inaugural total synthesis of (−)-crinipellin A was achieved by Lee’s group. A pivotal step in this synthesis involved the construction of the diterpene’s tetracyclic core via an intramolecular radical tandem [2 + 3] cycloaddition of an allenyl diazo substrate [33]. As delineated in Scheme 11, the synthesis commenced with commercially available compound **84**, which was converted to epoxyalkyne **85**. Subjecting epoxyalkyne **85** to an iron catalyzed S_N_2′ reaction with the requisite Grignard reagent, followed by silylation of the resultant secondary alcohol, yielded allene **86**. Subsequent acidic hydrolysis cleaved the acetal moiety in **86**, unmasking the aldehyde. Treatment of this aldehyde with *p*-toluenesulfonylhydrazide prepared hydrazone **87**, a perfect tandem cycloaddition precursor. Heating the sodium salt of the hydrazone triggered the conversion of **87** into diazo compound **88**, thereby initiating the formation of the tetracyclic framework. An ensuing intramolecular [2 + 3] cycloaddition between the diazo functionality and the allene group furnished methylenepyrazole intermediate **89**, which rapidly expelled nitrogen molecules to yield the transient TMM diyl species **90**. Despite considerable steric encumbrance, this highly reactive diyl intermediate underwent a final [2 + 3] dipolar cycloaddition with the olefin, affording the tetracyclic product **91**. Late-stage functional group modifications of compound **91** ultimately achieved the total synthesis of (−)-crinipellin A (**2**).

**Scheme 12:**
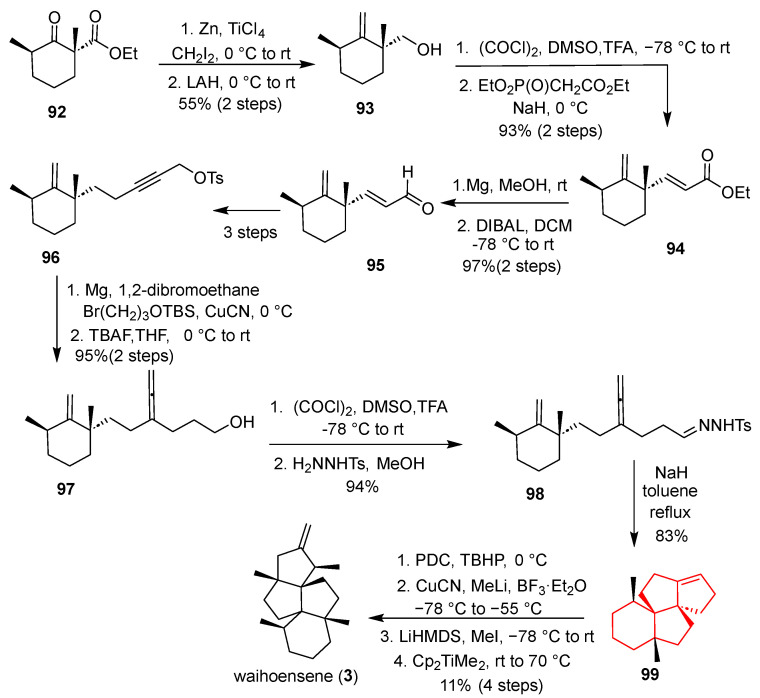
Synthesis of waihoensene by Lee’s group [34].

In 2017, Lee’s group achieved the total synthesis of waihoensene by employing a [2 + 3] cycloaddition approach consistent with earlier methodologies shown in Scheme 11. The synthesis of the cycloaddition precursor **98** was depicted in Scheme 12 [34]. Beginning with racemic ketoester **92**, a Lombardo–Takai olefination was performed, and the resulting product was reduced to give alcohol **93**. Oxidation under Swern conditions followed by a Horner–Wadsworth–Emmons olefination yielded α, β-unsaturated ester **94**, which was then selectively reduced using magnesium in methanol to afford aldehyde **95**. A three-step sequence comprising a Corey–Fuchs reaction, in situ hydroxymethylation, and tosylation delivered the functionalized propargylic alcohol **96**. This intermediate was transformed into allenyl alcohol **97** via a copper(I)-catalyzed S_N_2′ reaction and subsequent desilylation. Subjecting hydrazone **98** to a tandem [2 + 3] cycloaddition reaction constructed the core skeleton **99**, which underwent late-stage functionalization in four additional steps to complete the synthesis of waihoensene.

**Scheme 13:**
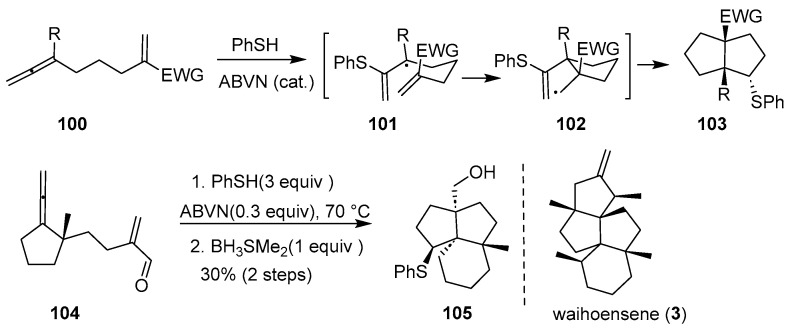
Synthesis of key skeleton of waihoensene by Yang’s group [35].

**Scheme 14:**
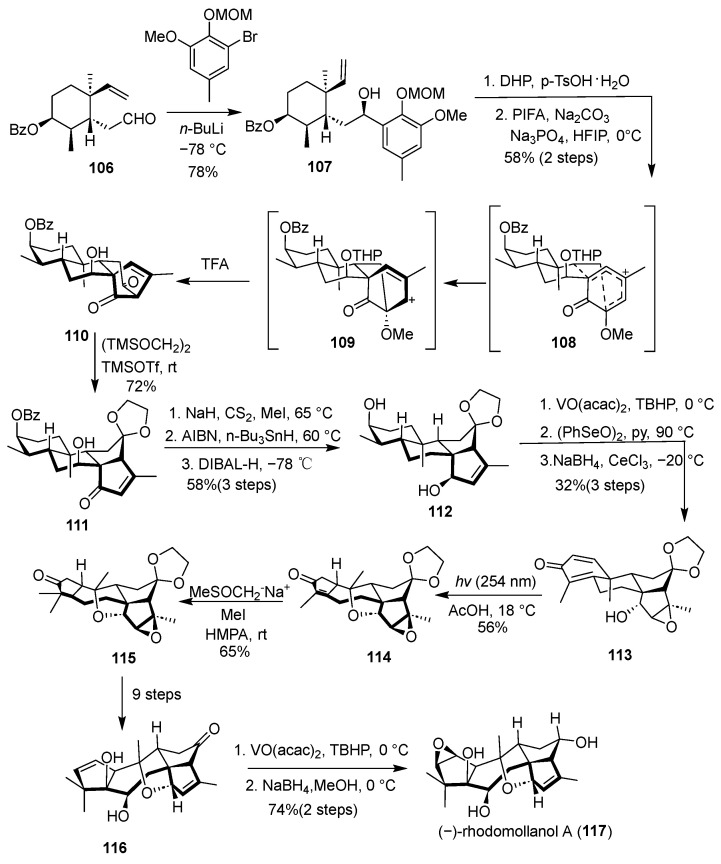
Synthesis of (–)-rhodomollanol A by Ding’s group [37].

In the same year, Yang’s group developed a benzenethiol-mediated cascade reaction involving intramolecular [3 + 2] cycloaddition between an allene and an α,β-unsaturated carbonyl compound, leading to the diastereoselective construction of a [3.3.0] bicyclic system featuring two quaternary bridgehead carbon atoms (Scheme 13) [35]. Using substrate **100**, the electrophilic benzenethiol radical was added regioselectively to the central sp-hybridized carbon of the allene, forming the stabilized tertiary radical **101**. This intermediate underwent 5-exo-trig cyclization onto the α, β-unsaturated system to give adduct **102**, which then participated in a 5-endo-trig cyclization, yielding a nucleophilic carbon-centered radical. Hydrogen abstraction from PhSH furnished product **103**—bearing vicinal quaternary centers at the bridgeheads—and regenerated the benzenethiol radical. Inspired by this efficient strategy for assembling diquinanes with two adjacent bridgehead quaternary stereocenters, the group extended the methodology to the synthesis of angularly fused triquinanes containing similar structural motifs. Accordingly, substrate **104** was successfully transformed into the corresponding triquinane **105,** with a 30% yield.

(–)-Rhodomollanol A, a natural product featuring a highly complex [3.5.7.5.5] hexacyclic framework with a distinctive 7-oxabicyclo [4.2.1] nonane core and eleven contiguous stereocenters, was first isolated from the Rhododendron species by Yao’s group in 2017 [36]. In 2020, Ding et al. accomplished the first asymmetric total synthesis of this structurally challenging molecule, as outlined in Scheme 14 [37]. Their synthesis commenced with compound **107**, which, upon oxidation with PIFA, underwent a one-step ODI-[5 + 2] cyclization tandem rearrangement to directly assemble the tetracyclic dicarbonyl compound **108**. This intermediate was then subjected to a tandem reverse Dieckmann cleavage/insertion–Dieckmann condensation sequence, effectively reorganizing the molecular skeleton to afford tricyclic ketone **110**. Subsequent elaboration of **110** over seven steps yielded cyclohexanedione **113**, which was further transformed via a light-promoted tandem Yamaguchi rearrangement/intramolecular etherification reaction to give ketone **114**, thereby establishing the core structure of the natural product. Finally, ketone **115** was advanced through an additional eleven steps to complete the inaugural asymmetric total synthesis of (–)-rhodomollanol A (**117**) from resveratrol-derived diterpenoid precursors.

**Scheme 15:**
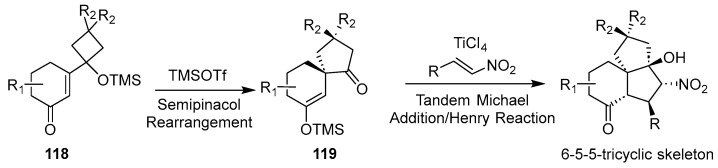
Synthesis of 6-5-5-tricyclic skeleton by Tu’s group [38].

In 2020, Tu and co-workers disclosed a novel one-pot cascade reaction sequence involving semi-pinacol rearrangement, Michael addition, and Henry reaction [38]. This transformation, catalyzed cooperatively by the dual Lewis acid system TMSOTf/TiCl_4_, utilized vinylogous α-ketols and nitroolefins as substrates to efficiently construct synthetically challenging 6-5-5 and 7-5-5 fused tricyclic carbocyclic frameworks. The methodology confers high structural complexity, generating products featuring up to five contiguous stereogenic centers, including a quaternary carbon stereocenter, within a convergent sequence. As shown in Scheme 15, the ketene compound **118** underwent a semi-pinacol rearrangement reaction under the action of Lewis acid TMSOTf to generate intermediate **119**. A Michael addition of compound **119** with the nitropropene compound under titanium tetrachloride conditions, and a subsequent Henry reaction, closed the last ring. This process featured a broad scope of substrates, providing a novel and efficient method for the synthesis of natural products containing this skeleton.

**Scheme 16:**
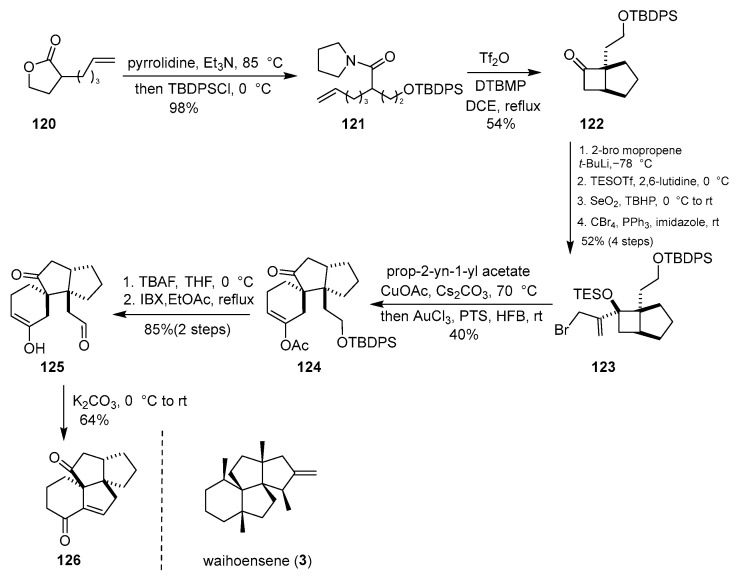
Synthesis of 6-5-5-tricyclic skeleton by Tu’s group [39].

In the same year, Tu and colleagues reported a continuous cascade process comprising Castro–Stephens coupling, 1,3-acetate migration, cyclization, and hemipinacol rearrangement for the construction of functionally rich spirocyclic [4.5] decanes from readily accessible starting materials [39]. This strategy was applied to the skeletal synthesis of the diterpenoid waihoensene. As illustrated in Scheme 16, commencing from compound **120**, allylic bromide **123** was synthesized over six steps, including a [2 + 2] cycloaddition and an aldol addition. Treatment of **123** with cuprous acetate and cesium carbonate afforded the tricyclic intermediate **124**, with a 40% yield, efficiently assembling the core framework. Late-stage manipulation, including an intramolecular aldol condensation, then delivered the tetracyclic core **126** of waihoensene (**3**).

**Scheme 17:**
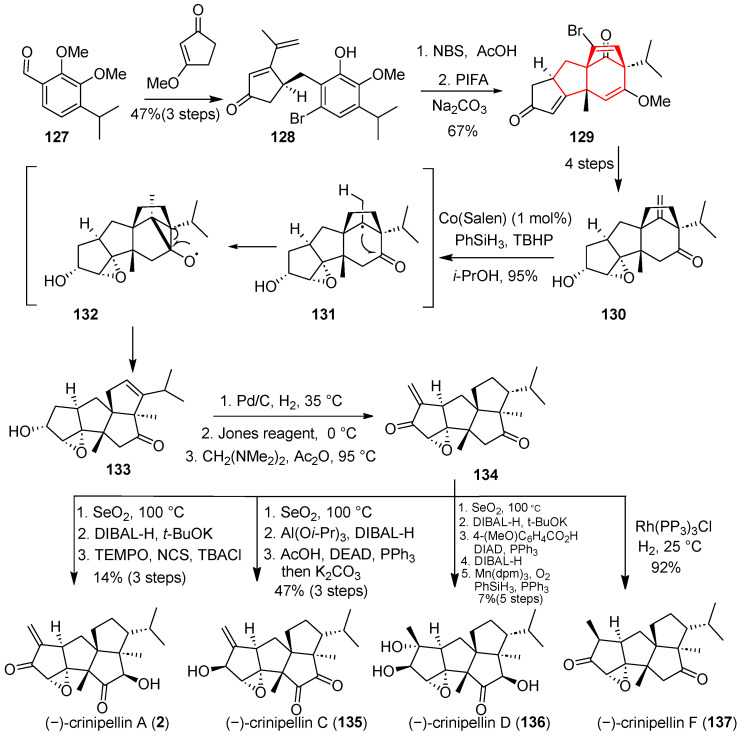
Synthesis of (−)-crinipellins by Ding’s group [40].

In 2022, Ding and co-workers disclosed a novel HAT-initiated Dowd–Beckwith rearrangement, enabling efficient access to diversely functionalized polyquinane scaffolds [40] (Scheme 17). Their synthetic approach featured an iridium-catalyzed regio- and enantioselective hydrogenation, followed by a diastereoselective ODI-[5 + 2] cycloaddition/pinacol rearrangement cascade. Starting from multisubstituted benzaldehyde **127**, condensation, asymmetric hydrogenation, and bromination afforded the key brominated intermediate **128**. Subsequent oxidation with hypervalent iodine reagent (PIFA) triggered an ODI-[5 + 2] cyclization and rearrangement sequence, smoothly delivering the tetracyclic framework **129**. Four additional steps converted **129** into olefin **130**, which then underwent an intramolecular Dowd–Beckwith rearrangement mediated by Co(Salen) and PhSiH_3_ to yield **133**. This critical rearrangement transformed the original [3.2.1] bridged system into a 5-5 fused ring skeleton. Notably, leveraging intermediate **134**, the team accomplished concise and collective asymmetric total syntheses of eight members of the Crinipellin family, highlighting the efficiency and versatility of this strategy.

**Scheme 18:**
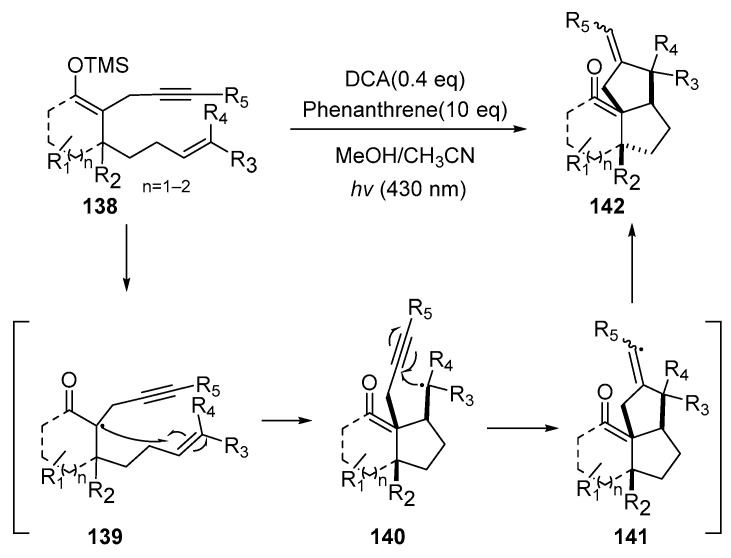
Synthesis of tricyclic skeleton by Liu’s group [41].

In 2023, Liu’s research group reported a photo-promoted enol silyl ether radical tandem cyclization reaction, which efficiently constructed a tricyclic framework compound [41]. As shown in Scheme 18, they used enol silyl ether **138** as the substrate and, under light conditions, first generated radical species **139** at the carbonyl ortho position. This radical underwent a 5-exo-trig cyclization, generating tertiary carbon radical **140**, which cyclized alkynes in the 5-exo-dig form, thus constructing skeleton compound **141** containing tricyclic rings. This method exhibited significant synthetic versatility, delivering products with exceptional stereoselectivity across diverse substrates.

**Scheme 19:**
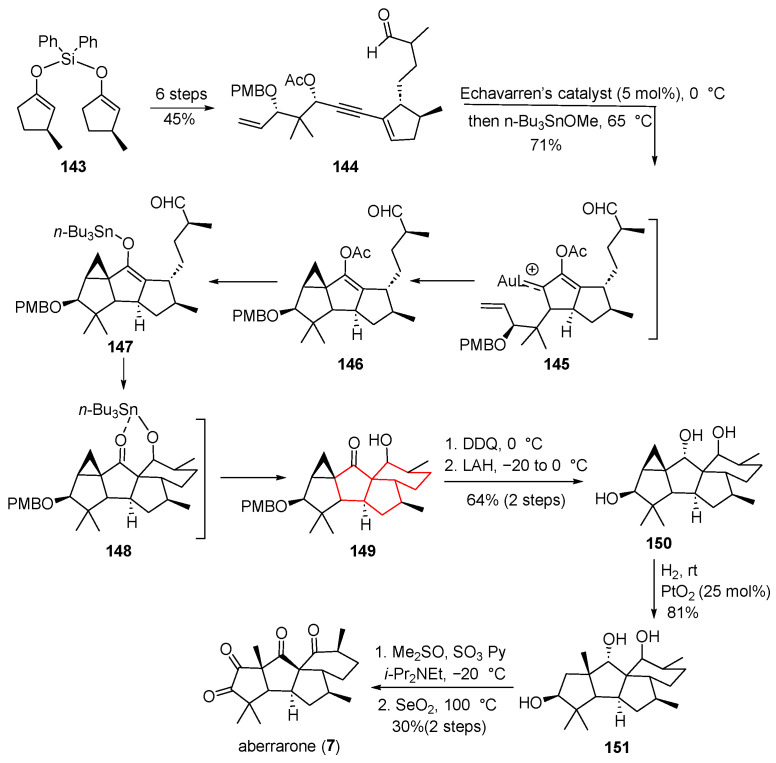
Synthesis of aberrarone by Carreira’s group [42].

In 2009, Rodríguez and colleagues isolated (+)-aberrarone from the Caribbean willow coral Pseudopterogorgia elisabethae; biological evaluation revealed potent in vitro anti-malarial activity [12]. Structurally, (+)-aberrarone possesses a distinctive 6-5-5-5 fused ring system incorporating four carbonyl groups and seven stereocenters, three of which are all-carbon quaternary centers. The first asymmetric total synthesis of this complex natural product was achieved by Carreira’s group in 2022 [42], pivoting on a Au/Sn-catalyzed Meyer–Schuster–Nazarov/cyclopropanation/aldol addition tandem sequence. As delineated in Scheme 19, the synthesis began with chiral enol silyl ether **143**, which was elaborated over six steps—including a MeLi-promoted aldol addition and Barton–McCombie deoxygenation—to furnish alkyne **144**. Exposure of **144** to Echavarren’s catalyst induced Meyer–Schuster rearrangement, generating allenol **146**. Subsequent treatment with *n*-Bu_3_SnOMe at 65 °C promoted cyclopropanation and aldol addition, delivering the pivotal pentacyclic intermediate **149** as a single diastereomer in 71% yield. Oxidative removal of the PMB group in **149**, followed by reduction with LAH, afforded triol **150**. Hydrogenation over PtO_2_ yielded **151**, which was then oxidized under Parikh–Doering conditions to the corresponding triketone. Finally, Riley oxidation using SeO_2_ completed the asymmetric total synthesis of (+)-aberrarone (**7**).

**Scheme 20:**
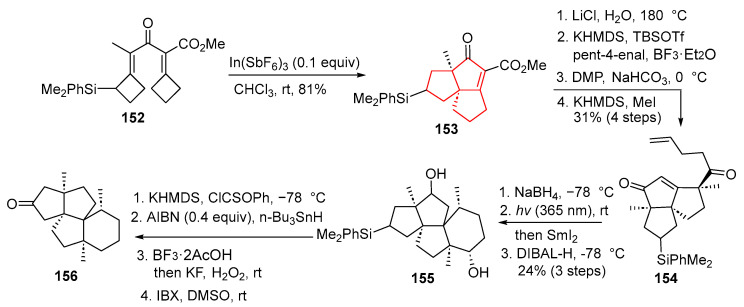
Synthesis of waihoensene by Tu’s group [43].

In 2022, Tu and colleagues established a modular and efficient route to angular triquinane-type frameworks containing quaternary carbon centers [43]. Their approach employed 1,3-dicyclobutylidene ketone (**152**) as a precursor, engaging it in an innovative cascade process consisting of a Nazarov cyclization and two successive ring expansions. As illustrated in Scheme 20, under In(SbF_6_)_3_ catalysis, compound **152** underwent cyclization to deliver the key tricyclic intermediate **153**. Subsequent decarboxylation, followed by a critical vinylogous aldol addition, Dess–Martin oxidation, and regioselective methylation, afforded enone **154**. This intermediate was then selectively reduced to generate a substrate suitable for a photoinduced intramolecular [2 + 2] cycloaddition. The resulting cycloadduct was subjected to SmI_2_-mediated ring-opening, yielding diol **155**. Finally, Barton–McCombie deoxygenation, Tamao–Fleming oxidation, and IBX oxidation completed compound **156**, which was finally transformed into (±)-waihoensene (**3**) following known procedures [25]. This synthesis highlights the power of a tandem cyclization–expansion cascade to rapidly build complex angular triquinane systems under mild conditions.

**Scheme 21:**
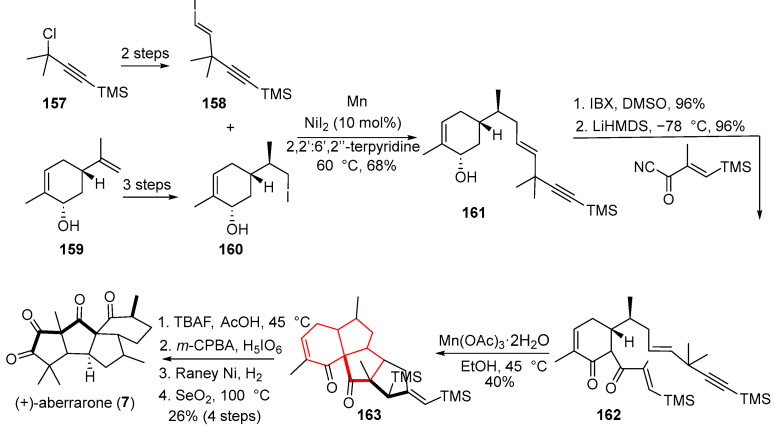
Synthesis of aberrarone by Jia’s group [44].

In 2023, Jia and colleagues accomplished a protecting-group-free total synthesis of the natural product (+)-aberrarone [44] (Scheme 21). A pivotal radical cascade cyclization enabled the simultaneous formation of three carbon–carbon bonds in a single operation, constructing three rings while precisely installing four new stereocenters. The synthesis commenced from commercially available (S,S)-carvacrol (**159**), which was converted to alkyl iodide **160** via a three-step sequence. A nickel iodide-catalyzed coupling between **160** and **158** then afforded compound **161**. Subsequent oxidation and acylation yielded radical precursor **162**. Treatment of this 1,3-dicarbonyl compound with manganese (III) acetate triggered an intramolecular 5-exo/5-exo/5-exo radical cascade, delivering the polycyclic product **163** in 40% yield. Further functionalization of **163** completed the total synthesis of (+)-aberrarone (**7**).

**Scheme 22:**
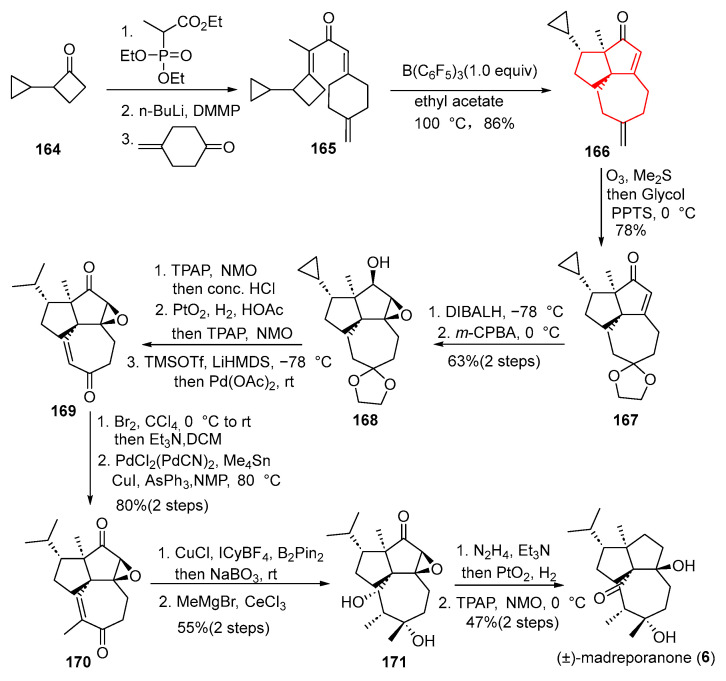
Synthesis of madreporanone by Tu’s group [45].

In 2024, Tu’s group developed a cascade Nazarov cyclization/dicycloexpansion reaction for the precise synthesis of the angularly fused tricyclic skeletons and executed a succinct total synthesis of madreporanone for the first time [45]. As shown in Scheme 22, the synthesis featured a pivotal, optimized cascade Nazarov cyclization/dicycloexpansion reaction of dienone **165** (prepared via Horner–Wadsworth–Emmons reactions from **164**) to construct the angular 5/5/7 tricyclic core (**166**) bearing vicinal quaternary stereocenters using B(C_6_F_5_)_3_ catalysis. Ozonolysis of compound **166**, followed by protection, led to acetal **167**. Selective reduction with DIBAL-H and epoxidation with m-CPBA afforded epoxide **168** diastereoselectively. Ley–Griffith oxidation and deprotection yielded a diketone, which underwent hydrogenation to remove an isopropyl group. A second Ley–Griffith oxidation and Saegusa–Ito oxidation then provided enone **169**. Bromination and Stille coupling installed a methyl group, yielding **170**. Subsequent 1,4-boronation/oxidation gave β-hydroxy ketone, which was converted to alcohol **171** via CeCl_3_-mediated MeMgBr addition. Wharton transposition and hydrogenation afforded triol, and final oxidation yielded madreporanone (**6**).

**Scheme 23:**
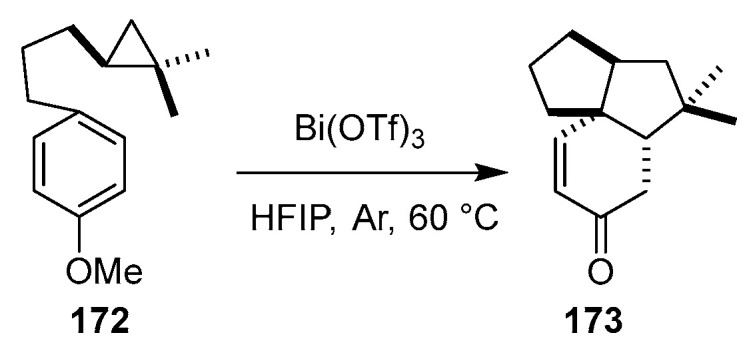
Synthesis of angular triquinanes by Wang’s group [46].

In 2024, Wang’s group reported a Lewis acid-catalyzed dearomative (3 + 2) intramolecular parallel cycloaddition (IMPC) strategy to construct angular 5-5-6 tricyclic cores relevant to natural products like norascyronone A, dankasterone A, and emervaridone A [46]. As shown in Scheme 23, the pivotal step employed donor–acceptor cyclopropanes (DACPs) tethered to benzene rings (e.g., 172). Under optimized conditions [Bi(OTf)_3_ (20 mol%), HFIP, 60 °C, 2–12 h], compound **172** underwent ipso-Friedel–Crafts-initiated ring-opening followed by Michael addition, efficiently forging the angular tricyclic skeleton (e.g., 173) with an embedded cyclohexenone.

Radical- or cation-initiated cascades achieve unparalleled step economy by assembling multiple rings/quaternary centers in a single operation. The “zipper effect” minimizes intermediate isolation, thereby improving overall yields. Photoredox variants enable chemoselective C–C bond formation under mild conditions. Cascade efficiency hinges on precise radical/ion stability gradients, often requiring tailored substrates. Diastereocontrol is compromised when multiple stereocenters form concurrently. Strict exclusion of protic impurities and stoichiometric radical mediators (Bu_3_SnH) impede industrial translation.

## 5. Emerging Frontiers: Photochemical and Ring-Expansion Tactics

**Scheme 24:**
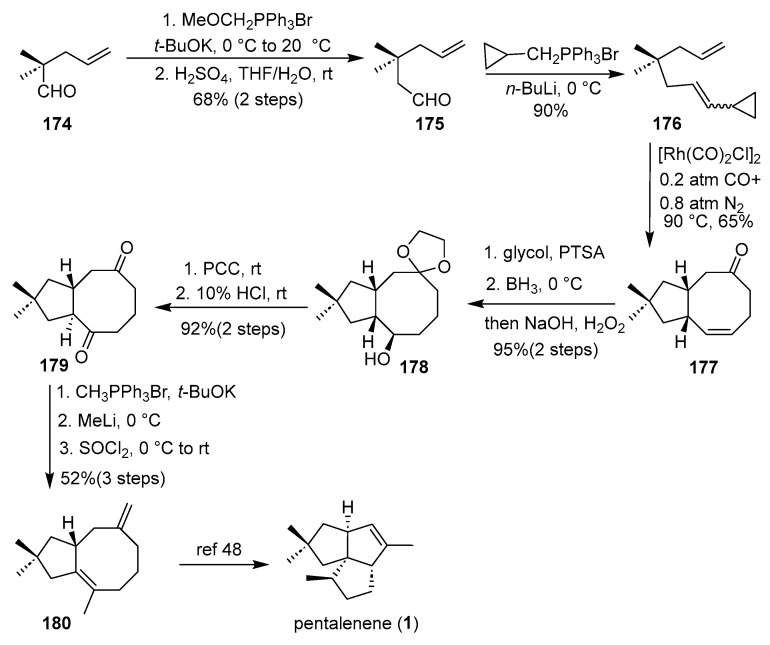
Synthesis of (±)-pentalenene by Yu’s group [47].

In 2009, Yu’s group achieved a formal synthesis of the angular triquinane natural product (±)-pentalenene using a Rh(I)-catalyzed [(5 + 2) + 1] cycloaddition as the pivotal step [47]. As shown in Scheme 24, the synthesis commenced with ene-vinylcyclopropane substrate **176**, which underwent cyclization under [Rh(CO)_2_Cl]_2_ catalysis in dioxane under a mixed CO/N_2_ atmosphere to furnish the bicyclic cyclooctenone **177**. This key intermediate was subsequently transformed through a sequence involving ketalization, hydroboration–oxidation (BH_3_·THF, then H_2_O_2_/NaOH), oxidation with PCC, and acid-mediated isomerization (10% HCl) to yield the advanced trans-diketone **179**. Further elaboration was carried out via Wittig olefination, methylation with MeLi, and dehydration using SOCl_2_/pyridine afforded diene **180**—a known precursor to pentalenene (**1**) [48]. This strategy highlighted the power of transition metal-catalyzed higher-order cycloadditions to rapidly build complex polycyclic cores, though the route was somewhat hampered by moderate regioselectivity in hydroboration and a modest overall yield.

**Scheme 25:**
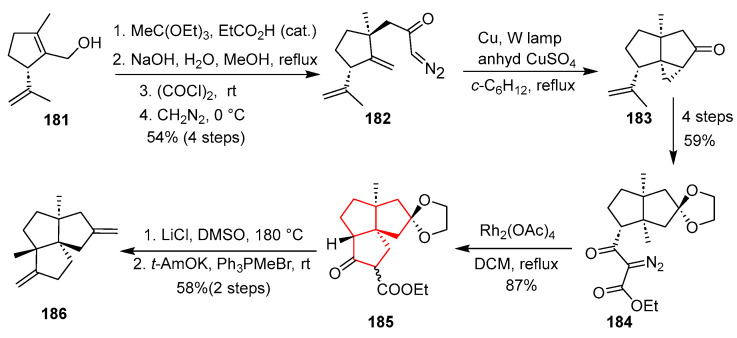
Synthesis of the angular triquinane core by Srikrishna’s group [49].

In 2011, Srikrishna and colleagues developed an enantiosecific route to angular triquinane natural products starting from the allylic alcohol **157**, readily available from (R)-limonene [49] (Scheme 25). The synthesis proceeded via Johnson orthoester Claisen rearrangement of **181** with triethyl orthoacetate to install the first quaternary center, yielding diazoketone **182**. Subsequent copper-catalyzed intramolecular cyclopropanation furnished the tricyclic ketone **183**, which underwent regioselective cyclopropane cleavage, ketalization, and ozonolysis, leading to the α-diazo-β-keto ester **184**. Treatment with Rh_2_(OAc)_4_ in refluxing CH_2_Cl_2_ effected highly regioselective intramolecular insertion into the tertiary methyl group, directly constructing the angular triquinane framework in **185**. Further steps including Krapcho decarbalkoxylation and double Wittig olefination yielded the advanced diene **186**, common to norsilphiperfolane and norcameroonane. This strategy showcased a powerful, stereocontrolled assembly of congested triquinanes via transition metal-catalyzed C–H functionalization, though the multi-step sequence and need for stereospecific starting materials may limit broad applicability.

**Scheme 26:**
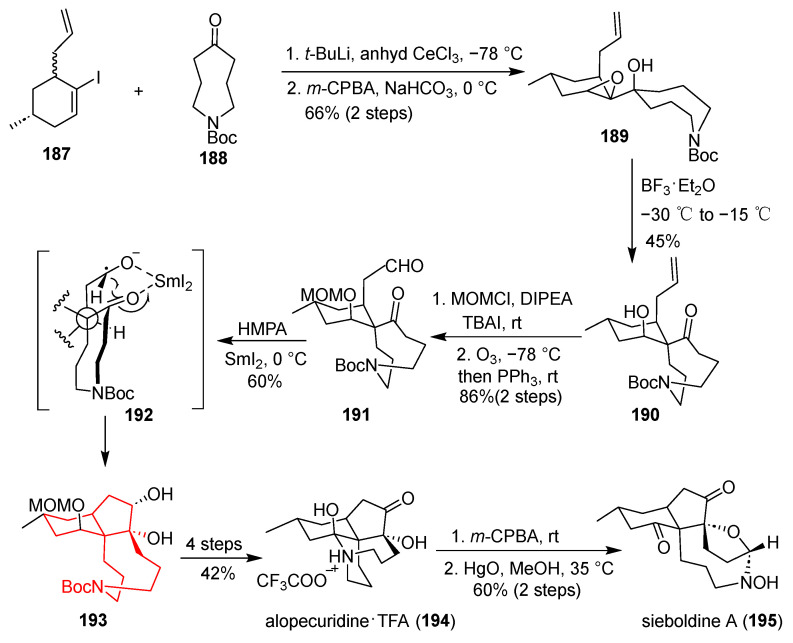
Synthesis of sieboldine A by Tu’s group [50].

In 2011, Tu’s group accomplished the first total synthesis of the complex alkaloid (±)-sieboldine A, featuring an angular triquinane-type core, via a biomimetic route from alopecuridine [50]. As shown in Scheme 26, the synthesis commenced with the coupling of iodoalkene **187** and carbamate **188** to form advanced intermediate. This was followed by stereoselective epoxidation with m-CPBA to furnish epoxy alcohol **189**. The pivotal semipinacol rearrangement of **189**, mediated by BF_3_·Et_2_O in diethyl ether at −30 to −15 °C, constructed the aza-cyclononane ring and established the all-carbon quaternary center, yielding key intermediates **190**. Subsequent MOM protection, ozonolysis, and a SmI_2_-promoted pinacol coupling forged the oxa-quaternary center and closed the B ring, delivering the tricyclic core **193**. Further functional group manipulations, including deprotection, oxidation (Ley oxidation), and Boc cleavage, afforded alopecuridine·TFA (**194**), which underwent biomimetic oxidation with m-CPBA and HgO to introduce the N-hydroxy group and form the tetrahydrofuran ring, completing the synthesis of sieboldine A (**195**). This route demonstrated the innovative use of semipinacol and pinacol couplings to efficiently assemble highly congested polycyclic architectures with contiguous quaternary centers.

**Scheme 27:**
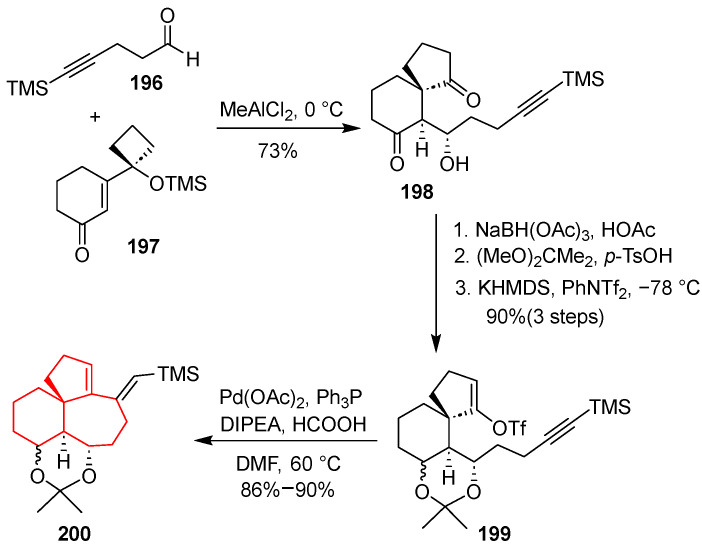
Synthesis of tricyclic skeleton by Tu’s group [51].

In 2012, Tu’s group developed a concise synthetic route to the [5,6,7] all-carbon tricyclic core characteristic of Calyciphylline A-type alkaloids, utilizing a tandem semipinacol-type 1,2-carbon migration/aldol reaction as the key transformation [51]. The synthesis commenced with the Lewis acid-mediated coupling of aldehyde **196** and trimethylsilane-protected vinylogous α-ketol **197** using MeAlCl_2_ in CH_2_Cl_2_ at 0 °C, which afforded 6-substituted spiro [4.5]decane-1,7-dione **198**. This one-pot tandem process efficiently established the spirocyclic quaternary center. The resulting diketone **198** was then subjected to selective reduction with NaBH(OAc)_3_ in acetic acid and protection with 2,2-dimethoxypropane, followed by enol triflation with PhNTf_2,_ resulting in enol triflate **199**. Palladium-catalyzed reductive cyclization using Pd(OAc)_2_ successfully constructed the angular [5,6,7] tricyclic framework in **200** with high efficiency. This strategy demonstrated a powerful and rapid approach to access highly congested polycyclic architectures via a novel tandem rearrangement–aldol sequence, enabling efficient installation of the spiro center and subsequent ring fusion (Scheme 27).

**Scheme 28:**
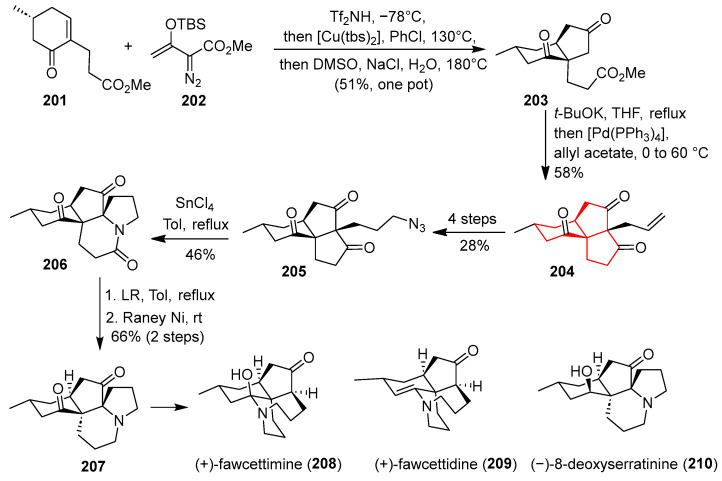
Synthesis of fawcettimine by Tu’s group [52].

In 2013, Tu’s group accomplished an efficient and divergent synthesis of the fawcettimine-class alkaloids, including the angular triquinane-containing (+)-fawcettimine, starting from chiral enone **201** and vinyl diazoacetate **202** [52]. As shown in Scheme 28, the synthesis commenced with a Mukaiyama–Michael addition and an intramolecular carbene addition/cyclization mediated by [Cu(tbs)_2_] in chlorobenzene at 130 °C, followed by decarboxylation, which furnished the cis-bicyclic dione **203** in a one-pot process. Subsequent Dieckmann condensation with *t*-BuOK and Tsuji–Trost allylation with allyl acetate constructed the 6/5/5 tricyclic system in **204**. After protection of the ketone, hydroboration–oxidation and Mitsunobu azidation provided azidotrione **205**. The regioselective intramolecular Schmidt reaction of **205** using SnCl_4_ in refluxing toluene effected N-insertion to form lactam **206**, which was converted via thionation and reduction into the common intermediate **207**. Finally, three members of fawcettimine (**208**, **209**, **210**) were synthesized. This route exemplifies a highly convergent and modular strategy for constructing congested angular triquinane frameworks, leveraging powerful metal-catalyzed cyclizations and rearrangement reactions to achieve brevity (ten steps total) and functional group compatibility.

**Scheme 29:**
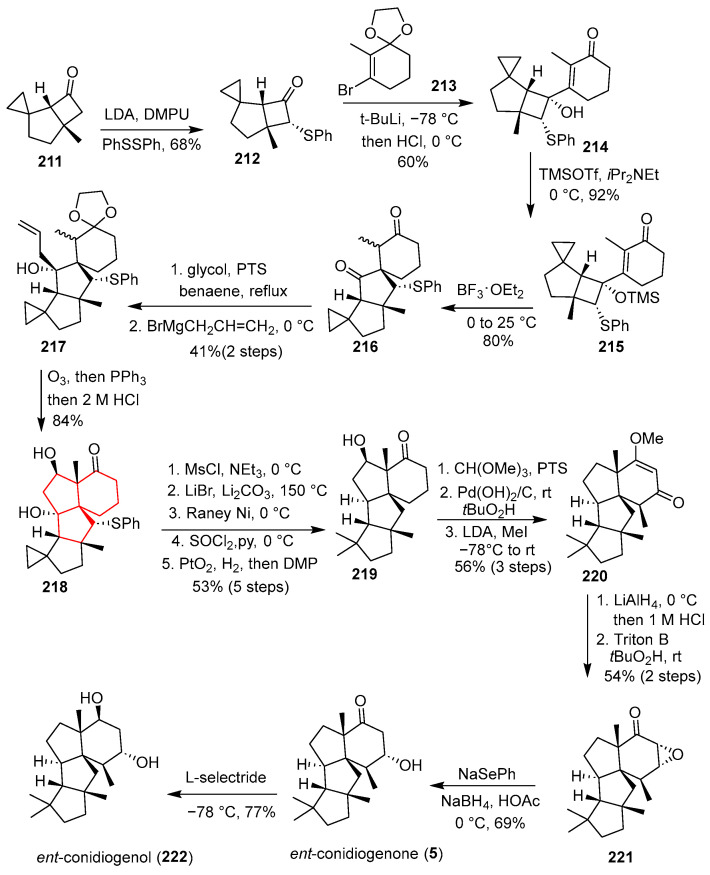
Synthesis of conidiogenol by Tu’s group [53].

In 2016, Tu’s group reported the first total synthesis of the cyclopiane-type tetracyclic diterpenes conidiogenone and conidiogenol [53] (Scheme 29). Their synthetic approach commenced with the coupling of vinyl bromide **213** and the phenylthio-substituted cyclobutanone **212** via lithium–halogen exchange followed by nucleophilic addition, yielding the key tertiary alcohol intermediate **214**. This was subsequently protected to form the rearrangement precursor **215**. The cornerstone of their strategy was a Lewis acid-mediated semipinacol-type cycloenlargement, specifically using BF_3_·OEt_2_ in CH_2_Cl_2_, which effectively constructed the spirocyclic A/C ring system with the desired regioselectivity and diastereoselectivity, affording compound **216**. This critical step established the angular triquinane core bearing the requisite quaternary centers. Compound **216** was subjected to a ketal protection and chiral resolution sequence, followed by a diastereoselective allylation with allylmagnesium bromide to afford alcohol **217** as a single diastereomer. Ozonolysis of the alkene in **217** generated an aldehyde, which underwent spontaneous intramolecular aldol cyclization under acidic conditions to construct the pentacyclic framework in **218** with high stereocontrol. Further functional group manipulations, including elimination, hydrogenation, and oxidation, provided ketone **219**. Compound **220** was then transformed into epoxide **221**, and subsequent reductive ring-opening with NaSePh afforded conidiogenone (**5**). Finally, reduction of **5** with L-selectride produced conidiogenol (**222**). The synthesis featured a highly efficient and stereocontrolled assembly of the congested tetracyclic framework, though the necessity for chiral resolution and multiple steps to introduce and remove the phenylthio auxiliary slightly diminished the overall atom economy.

**Scheme 30:**
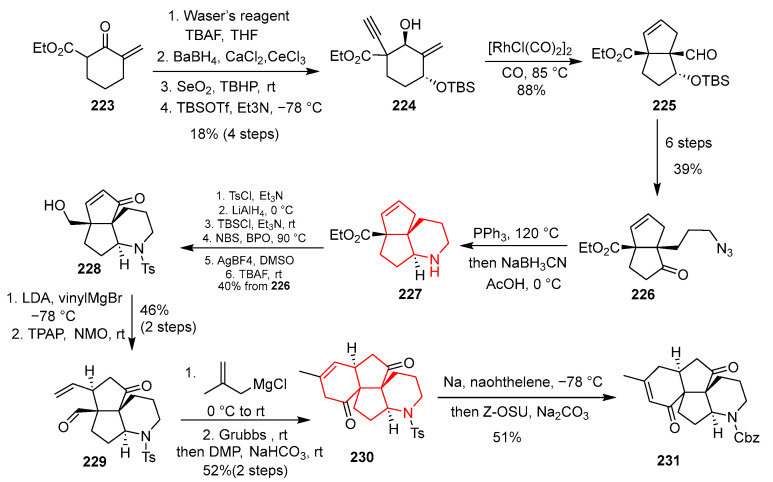
Synthesis of lycojaponicumin C by Yang’s group [54].

In 2017, Yang’s group developed a formal total synthesis of (±)-lycojaponicumin C, constructing its angular triquinane core through a highly strategic sequence [54] (Scheme 30). Starting from commercially available ethyl acetoacetate **223**, the key enyne precursor **192** was prepared via Waser alkynylation and Luche reduction on a decagram scale. The pivotal step involved a Rh(I)-catalyzed formal [3 + 2] cycloaddition of **224** under a CO atmosphere, efficiently forging the bicyclic [3.3.0] scaffold **225** with two adjacent all-carbon quaternary centers in excellent diastereoselectivity. Aldehyde **225** was then elaborated through a double-Wittig reaction, reduction, and mesylation-azidation to give keto-azide **226**. A Staudinger/reductive amination cascade on **226** constructed the piperidine ring, yielding intermediate **227**. Subsequent functional group manipulations, including tosylation, an ingenious γ-OH-directed vinyl Michael addition, and Ley oxidation, installed the requisite stereochemistry and functionality to deliver aldehyde **229**. Grignard addition with 2-methylallyl magnesium chloride then provided diene, which underwent ring-closing metathesis (RCM) and double bond migration to form the cyclohexanone ring, affording advanced intermediate **230** and completing the tetracyclic skeleton of the natural product. This synthesis is notable for its innovative use of transition metal catalysis to build sterically congested systems and directed reactions to control stereochemistry in highly hindered environments. However, the route involved multiple functional group interconversions and a late-stage RCM, which somewhat diminished its overall atom economy and step efficiency.

**Scheme 31:**
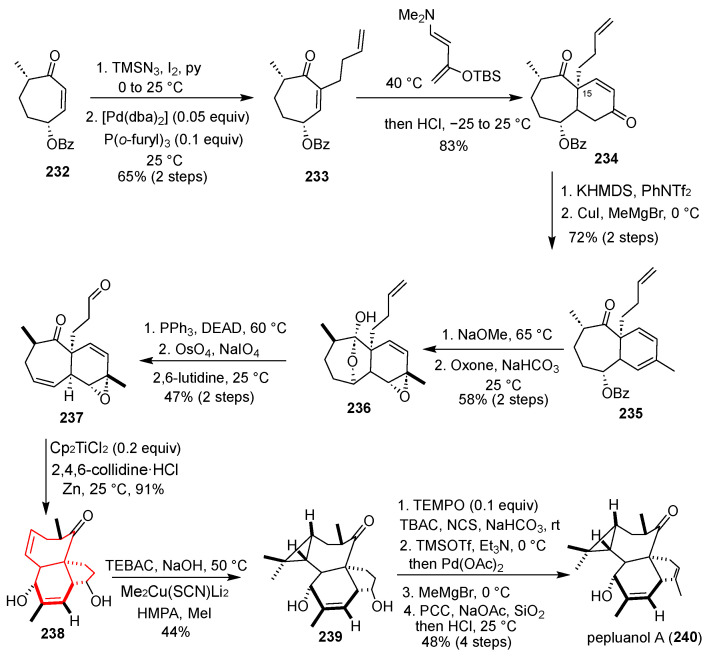
Synthesis of pepluanol A by Ding’s group [55].

Pepluanol A (**240**) was isolated from Euphorbia peplus, a plant in the *Euphorbia genus*, in 2016 with a [5,6,7,3] four-ring skeleton with seven chiral centers, including six continuously distributed centers and one quaternary carbon center. The synthesis of this molecule presents significant challenges. In 2017, Ding’s group achieved the first total synthesis of the complex Euphorbia diterpenoid (±)-pepluanol A, utilizing an innovative titanium(III)-catalyzed reductive annulation as a pivotal strategy [55] (Scheme 31). Their synthesis commenced with readily available cycloheptenone **232**, which was converted to enone **233** in two steps. A high-pressure Diels–Alder cycloaddition between **233** and the Rawal diene constructed the bicyclic framework **234**, introducing the crucial C15 quaternary carbon center as a single diastereomer. Subsequent functionalization, including regioselective enol triflation, methyl cuprate addition, and base-induced epimerization, yielded vinyl epoxide **236**. Dehydration and Lemieux–Johnson oxidation then afforded the key vinyl epoxide–aldehyde coupling partner **237**. The core transformation was a titanium(III)-catalyzed reductive annulation of **237** (Cp_2_TiCl_2_/Zn, collidine·HCl, THF), which efficiently forged the [5,6,7]tricyclic 1,5-diol **238**—the angular triquinane core precursor—in excellent yield, albeit with moderate diastereoselectivity. Late-stage synthesis involved a diastereoselective cyclopropanation via dibromocarbene addition to **238** and reductive bismethylation to assemble the tetracyclic skeleton **239**. Finally, a TEMPO/NCS oxidation, Saegusa–Ito dehydrogenation, regioselective Grignard addition, and a Dauben–Michno oxidation completed the synthesis of pepluanol A (**240**). This route is notable for its convergent design and the innovative, atom-economical reductive annulation to construct the challenging 1,5-diol moiety. However, the moderate diastereoselectivity in the key cyclization and the requirement for extensive functional group manipulations in the late stage present opportunities for further refinement.

**Scheme 32:**
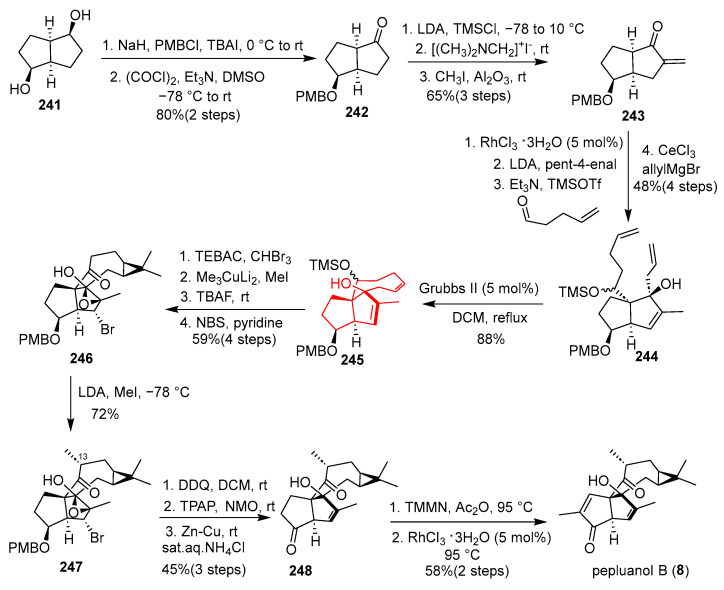
Synthesis of pepluanol B by She’s group [56].

Pepluanol B (**8**) is a structurally novel diterpene molecule isolated by Qiu’s group from Euphorbia Euphorbia in Southwest China [14,15]. Structurally, pepluanol B has a unique [5,5,8,3] four ring skeleton structure with six chiral centers (including a bridgehead all carbon quaternary carbon chiral center). In 2020, She’s group reported the first total synthesis of (–)-pepluanol B, which features a challenging [5-5-8-3] tetracyclic framework [56] (Scheme 32). The synthesis commenced with known bicyclic diol **241**, which was elaborated through PMB protection and oxidation to afford ketone **242**. Subsequent Eschenmoser methylenation provided exo-methylene intermediate **243**. Rh-catalyzed isomerization, aldol reaction, TMS protection, and CeCl_3_-mediated allylation obtained diene **244**. The pivotal ring-closing metathesis (RCM) of **244** using Grubbs II catalyst forged the eight-membered ring, delivering cyclic Z-alkene **245** as a key intermediate. Dibromocyclopropanation of **245** and reductive bismethylation then constructed the gem-dimethyl cyclopropane unit, yielding advanced intermediate **246**. Methylation with LDA proceeded smoothly, affording compound **247** with the established R configuration at the C13 stereocenter. Final Zn–Cu–mediated deprotection yielded allylic alcohol **248**, and α-methylenation followed by Rh-catalyzed isomerization completed the synthesis of pepluanol B (**8**). This route is notable for employing strategic C–C bond formations like aldol and RCM. However, the synthesis involves multiple functional group manipulations and sensitive intermediates, resulting in moderate overall yield, yet it provides efficient access to the complex angular triquinane framework and enables further biological studies.

**Scheme 33:**
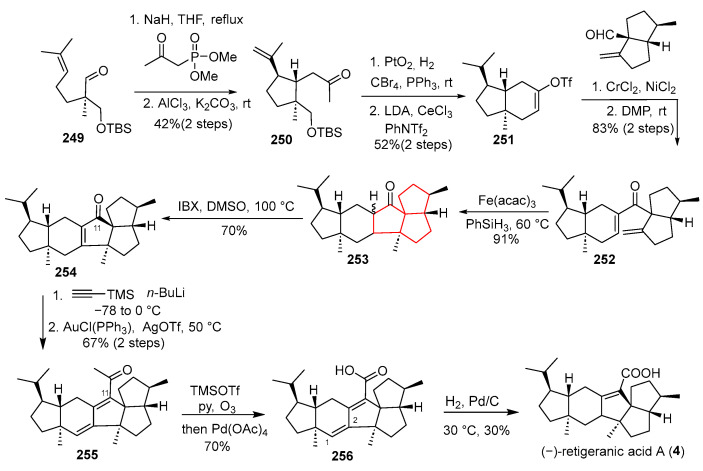
Synthesis of retigeranic acid A by Chen’s group [57].

In 2023, Chen’s group reported an enantiocontrolled total synthesis of (–)-retigeranic acid A, which features a strategic construction of the angular triquinane core [57] (Scheme 33). Beginning from aldehyde **249**, a Horner–Wadsworth–Emmons homologation and a diastereoselective Prins cyclization under AlCl_3_ catalysis furnished the trans-hydrindane framework **250**. Subsequent functional group manipulations, including hydrogenation and α-alkylation, converted **215** into vinyl triflate **251**. A Nozaki–Hiyama–Kishi coupling between **251** and aldehyde yielded ketone **252**. The pivotal angular triquinane formation was then attempted via an Fe-mediated hydrogen atom transfer (HAT)-initiated 5-endo-trig radical cyclization. The reaction yielded Paquette-type intermediate **253**. Oxidative dehydrogenation of **253** with IBX provided enone **254**. Introduction of the C11 carbon was achieved through addition of lithium (trimethylsilyl)acetylide, followed by hydrolysis to give methyl ketone **255**. A multi-step sequence involving enolization, ozonolysis, and Pb(OAc)_4_ oxidation then installed the C11 carboxylic acid, yielding diene acid **256**. The synthesis culminated in a high-pressure hydrogenation over Pd/C that reduced the C1–C2 double bond and epimerized several centers, delivering a mixture from which (–)-retigeranic acid A (**4**) was isolated. This route stands out for its innovative use of a Prins cyclization to assemble the trans-hydrindane system and a late-stage HAT-based radical cyclization to form the congested triquinane core.

**Scheme 34:**
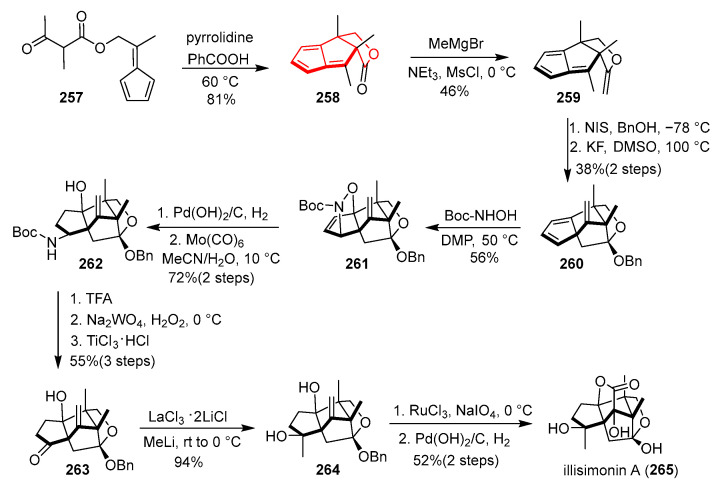
Synthesis of illisimonin A by Lu’s group [58].

In 2025, Lu’s group reported a gram-scale total synthesis of the complex sesquiterpenoid (±)-illisimonin A, featuring a novel strategy centered on higher-order cycloadditions for constructing its challenging caged pentacyclic framework [58]. As shown in Scheme 34, beginning with fulvene ester **257**, the key step involved an intramolecular [6 + 2] cycloaddition under pyrrolidine and benzoic acid catalysis at 60 °C, efficiently furnishing the stereochemically dense cis-fused 5/5/5 tricyclic lactone **258** with two adjacent quaternary centers in 81% yield. Subsequent conversion of **258** to enol ether **259** was achieved via methylation with MeMgBr followed by dehydration with MsCl/Et_3_N. The pivotal angular triquinane precursor was then assembled through an iodoetherification of **259** followed by an intramolecular alkylation to deliver cyclopentadiene **260**. A critical nitroso-Diels–Alder reaction with an in situ-generated acyl nitroso species afforded the stable nitroxide adduct **261** as the major thermodynamic product. Hydrogenation of **261** and Mo(CO)_6_-mediated N–O bond cleavage provided carbamate **262**. Deprotection of **262** with TFA, followed by sequential oxidation and TiCl_3_·HCl reduction, yielded ketone **263**. Diastereoselective methyl addition to **263** with MeLi/LaCl_3_·2LiCl delivered diol **264** with excellent selectivity. Finally, lactonization via RuCl_3_/NaIO_4_-mediated oxidative cyclization constructed the α-hydroxy lactone motif, and global deprotection completed the synthesis of (±)-illisimonin A (**265**). This route is notable for its innovative use of a pentafulvene-based [6 + 2] cycloaddition to rapidly build the polycyclic core and a nitroso-Diels–Alder reaction to overcome earlier peroxide instability issues, enabling a scalable and convergent synthesis in 14 steps.

Table 1 reveals nuanced trade-offs in waihoensene synthesis. Pauson–Khand approaches (Snyder, Yang, Gaich) excel in step efficiency (LLS 14–17) and yield (1.1–4.0%), with Gaich’s 14-step route leading at 4.0%. Their intramolecular Pauson–Khand reaction enables stereoselective tricyclic core construction (e.g., Snyder’s 50% yield) with inherent cis-fused control, leveraging atom economy. However, reliance on toxic Co_2_(CO)_8_ limits scalability, and substrate rigidity demands protective group manipulations. Tandem cyclization methods (Lee [34], Tu [43]) offer step economy—Lee’s one-pot tetracyclic assembly via [3 + 2] cycloaddition; Tu’s Nazarov/ring-expansion cascade—but suffer longer LLS (18) and lower yields (0.99–2.8%). Diastereocontrol challenges in concurrent stereocenter formation and regioselectivity issues in cascades hinder their performance, making Pauson–Khand strategies more robust for this target.

## 6. Conclusions

The synthesis of architecturally complex angular triquinane natural products continues to challenge and inspire synthetic chemists. This review highlights four dominant strategies: Pauson–Khand reactions (convergent, stereoselective, but limited substrate scope), tandem cyclizations (superior step economy, yet often compromised diastereocontrol), skeletal reorganizations (biomimetic potential, but demanding precise substrate design), and emerging methods (ring expansions, photochemistry). Collectively, these approaches have significantly advanced access to these strained frameworks. Future priorities must address (i) catalytic asymmetric variants of skeletal reorganizations; (ii) AI-guided retrosynthesis for polyquinane cores; and (iii) late-stage diversification to explore bioactivity diversity. The comparative analysis presented herein provides a strategic foundation for selecting and refining methodologies towards current and future targets.

## Figures and Tables

**Table 1:** Comparative analysis of synthetic strategies for waihoensene.

Synthetic Approach	Research Group	LLS	Overall Yield	Key Step	Source
Pauson–Khand	Snyder et al. (2020) [24]	17	1.1%	Intramolecular Pauson–Khand reaction (50% yield, constructs tricyclic core)	Scheme 3
	Yang et al. (2020) [25]	15	3.8%	Pauson–Khand reaction (moderate yield)	Scheme 4
	Gaich et al. (2021) [26]	14	4.0%	Pauson–Khand reaction	Scheme 5
Tandem Cyclization	Lee et al. (2017) [34]	18	0.99%	Tandem [3 + 2] cycloaddition of allenyl diazo (one-step tetracyclic skeleton assembly)	Scheme 12
	Tu et al. (2022) [43]	18	2.8%	Nazarov cyclization/double ring-expansion cascade (forms angular tricyclic core)	Scheme 20

## Data Availability

The research data are available by contacting the corresponding author.
